# Cancer risk in patients with pulmonary fibrosis and a rare telomere related gene variant

**DOI:** 10.1186/s12931-025-03469-2

**Published:** 2026-01-19

**Authors:** Joanne J. van der Vis, Martijn T. K. Maus, Charlotte I. de Bie, Jasper J. van der Smagt, Laura G. M. Daenen, Matthijs F.M. van Oosterhout, Jan C. Grutters, Coline H. M. van Moorsel

**Affiliations:** 1 ILD Center of Excellence, Member of European Reference Network-Lung, St Antonius Hospital, Nieuwegein, the Netherlands; 2 Department of Clinical Chemistry, St. Antonius Hospital, Nieuwegein, the Netherlands; 3 Department of Genetics, University Medical Center Utrecht, Utrecht, the Netherlands; 4 Department of Hematology, University Medical Center Utrecht, Utrecht, the Netherlands; 5 Department of Pathology, Pathologie DNA-BV, St Antonius Hospital, Nieuwegein, the Netherlands; 6 Division of Heart and Lungs, University Medical Center Utrecht, Utrecht, the Netherlands

**Keywords:** Pulmonary fibrosis, Telomere biology disorder, Genetic counselling, Cancer, Myelodysplastic syndrome

## Abstract

**Background:**

Rare telomere related gene (TRG) variants are the cause of telomere biology disorders (TBD), a broad spectrum of age-related diseases, such as pulmonary fibrosis (PF) a disease manifesting at older age. TBD patients are at increased risk of certain types of squamous cell carcinomas and hematological malignancies. However, most studies on cancer incidence in TBDs involved relatively young cohorts in which patients with pulmonary fibrosis were underrepresented or absent. Therefore, we investigated cancer incidence in patients with pulmonary fibrosis as major manifestation of TBD (TBD-PF) with a pathogenic (P), likely pathogenic (LP), and variant of unknown significance (VUS) TRG-variant and compared it to the general population.

**Methods:**

TBD-PF patients (*n* = 177) were retrospectively included and cancer history was collected. For each cancer type the observed number of cancers was compared with the expected number adjusted for age and sex based on data of the Netherlands Cancer Registry.

**Results:**

The TBD-PF cohort (median age 62.8 years, 63% male), consisted of 114 P/LP and 63 VUS carriers. Restricting the analyses to P/LP variant carriers we observed 14 patients with cancer (solid cancer *n* = 8 (7.0%); hematological cancer *n* = 6 (5.3%)). The observed all-cancer incidence (12.3%) was comparable to expected (18.9%) in the general population, Observed/Expected incidence 0.6 (95%CI 0.4-1.0). Regarding cancer types, only myelodysplastic syndrome was significantly more frequently observed than expected (Observed/Expected = 56 (95%CI 21–117)). There were no significant differences in clinical characteristics and transplant-free survival between TBD-PF patients with and without cancer. Comparable results were obtained when patients with TRG variants classified as VUS were included.

**Conclusion:**

All-cancer risk for TBD-PF patients was comparable to the general population. However, TBD-PF patients have an increased risk of developing myelodysplastic syndrome, underlining the importance of tailored genetic counselling and hematological surveillance.

**Supplementary Information:**

The online version contains supplementary material available at 10.1186/s12931-025-03469-2.

## Background

Pulmonary fibrosis (PF) is a rare aging disease caused by scarring of the lungs and manifests predominantly as idiopathic pulmonary fibrosis (IPF). This archetypical progressive form of PF has a median survival of 3–5 years [1–3] and up to 20% have familial PF [4, 5]. Familial pulmonary fibrosis (FPF) studies have identified rare telomere related gene (TRG) variants as the most common genetic cause [6, 7]. Carriers of rare TRG-variants have accelerated loss of telomere length resulting in critically short telomeres inducing cellular senescence and premature organ aging [8].

Telomere length is a heritable trait, and in families with rare TRG-variants, telomeres shorten in successive generations leading to disease anticipation [9–11]. TRG-variants cause not only PF, but a broad spectrum of age-related phenotypes, termed telomere biology disorders (TBDs) [12]. PF is often the first manifestation of a TBD in families with rare TRG variants [13] and mostly presents at older age [11, 14, 15]. Other forms of TBD usually manifest at an earlier age. In younger individuals, TBD associated bone marrow failure (TBD-BMF), for instance manifesting as dyskeratosis congenita (DC) is more common. Younger TBD patients are at high risk of aplastic anemia, myelodysplastic syndrome (MDS), acute myeloid leukemia (AML), and solid tumors, particularly head and neck squamous cell carcinoma (HNSCC) [16–18]. Accordingly, close monitoring of individuals with TBD-BMF is essential for timely interventions and cancer management [16].

With increasing knowledge on genetics and TBD, clinical implementations are emerging. Recent US and European statements advocate genetic analysis and counselling in cases with FPF [19, 20]. The genetic status of the patient informs diagnosis, and guides variant-associated risk management. Based on current knowledge, management of TBD-BMF and related TBD includes regular cancer screening [21]. Screening for specific tumors (leukemia, HNSCC, gynecologic-, rectal-, esophagus-, liver- skin- and lung-cancer) are advised by the TBD diagnosis and management guidelines [21, 22]. Therefore, in our clinic PF patients with a (likely) pathogenic TRG-variant are advised to regularly screen for oral cavity tumors and gynecologic squamous cell carcinoma.

These recommendations are largely based on expert opinion and TBD patient cohorts with a large proportion of relatively young (TBD-BMF) patients. In TBD, pulmonary fibrosis is the most common manifestation [23] typically diagnosed in the sixth or seventh decade of life with predominantly isolated lung involvement [11, 14, 15]. To date, the cancer risk and spectrum of patients with PF as major manifestation of TBD is not comprehensively investigated. Studies on cancer incidence in TBD have primarily focused on early-onset TBDs, such as DC and TBD-BMF, or included mixed TBD phenotypes [18, 24–26], while studies investigating older TBD patients with PF lack comparisons with the general population [11, 15, 27, 28]. Consequently, it remains unclear whether current cancer surveillance strategies for TBD patients are also applicable to the older TBD patients with PF as major manifestation.

In this study, we assess cancer incidence and risk in a large cohort of patients carrying rare TRG-variants, with PF as major TBD manifestation, in comparison with the general Dutch population. Additionally, we summarize and contextualize findings within the TBD-cancer literature.

## Methods

### Patients

At the St Antonius ILD Center of Excellence, genetic analysis is performed in patients who: (1) have a first- or second-degree family member with PF, (2) are diagnosed with PF before the age of 50 or (3) are suspected of having telomere biology disorders. Our cohort included 186 patients with PF and a rare TRG-variant who were diagnosed between March 2002 and September 2022 and were screened for eligibility for inclusion in the study. From 179 of the 186 patients, medical records were available for evaluation of history of cancer. Only patients in whom PF represented the major manifestation (that is, the predominant clinical feature) of TBD and a rare TRG-variant classified as pathogenic, likely pathogenic or variant of unknown significance, according to the American College of Medical Genetics and Genomics and the Association for Molecular Pathology recommendations [29] were included (table E1). Data was retrospectively collected from medical records between February 5 and November 17, 2023. Two patients had a dyskeratosis congenita (DC) diagnosis prior to the PF diagnosis, because the cancer risk and spectrum of patients with DC have been described, these patient were excluded. In total 177 rare TRG variant carrying patients with PF as major manifestation of TBD (177 TBD-PF) were included in the study. In the main body of the manuscript, analyses were restricted to TBD-PF patients carrying a rare TRG variant classified as likely pathogenic or pathogenic (*n* = 114). Results from the patients including variants of unknown significance are provided in the Supplementary Data file (table E1-E7) to enable a comprehensive overview of the genetic spectrum observed in TBD-PF and to facilitate potential future reclassification of rare telomere related gene variants. The Medical Research Ethics Committees United of the St. Antonius Hospital approved the ILD Biobank study (R05-08 A), and written informed consent was obtained from all participants.

### Clinical characteristics

The age at diagnosis of PF was documented at time of multidisciplinary team discussion when available, or during the first consult at the outpatient clinic of St Antonius ILD Center of Excellence. Demographics, smoking status and cancer history were retrospectively collected from patient records. A positive history for cancer included documented ever presence of a solid tumor or hematologic malignancy. MDS diagnoses had been established between April 2008 and October 2018, based on the diagnostic criteria and knowledge available at that time, and in alignment with the methodology of previous studies [16, 26] MDS diagnosis were documented by clinical hematologists and referral letters in the medical records of the patient. Telomere length was measured in DNA from total leukocytes in peripheral blood by quantitative PCR, detailed information is provided in the supplementary file.

### Statistical analysis

The observed cancer incidence was calculated as the number of patients with both PF and cancer divided by the total number of patients with PF. For each cancer type the observed number was compared with the expected number based on the numbers reported in the Netherlands Cancer Registry (NCR) in 2019, reference date November 2023 [30]. Sex adjustment was performed by using sex-specific crude incidence rates for males and females separately. Age adjustment was performed by calculating the cumulative incidence for each individual patient up to the age of last contact or age of death. The cumulative incidence (CuI) for each patient was calculated with the formula: CuI = 1–EXP(-IR*T) (IR = crude rate incidence adjusted for sex from the NCR; T = age at last contact or death in years). To calculate the expected number of cancer for the PF patient cohort, the CuI from all patients were aggregated and divided by the total number of PF patients in the study (*n* = 177). In this study we aimed to evaluate whether TBD-PF patients require additional cancer screening during their lifetime, therefore we have chosen for the current approach which appropriately reflects the clinically relevant risk of cancer during life in this specific patient population. More details are provided in the supplementary file.

## Results

### Cohort description

**Fig. 1:**
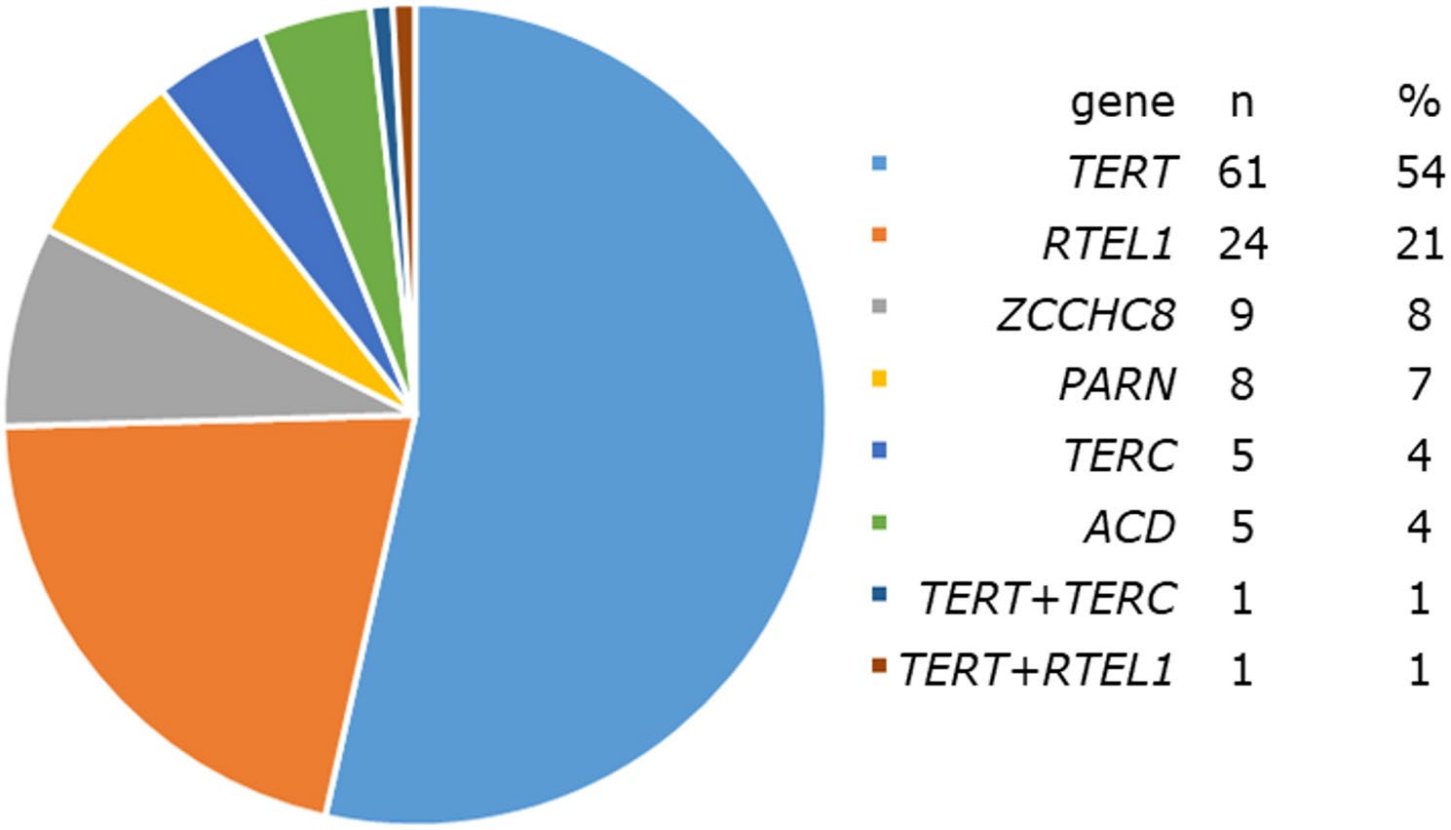
Spectrum of mutated telomere related genes in patients with pulmonary fibrosis

The TRG-variant carrying cohort consisted of 177 TBD-PF patients from 113 families, with 1 to 6 patients per family. The primary analysis was restricted to patients carrying TRG variants classified as P/LP (*n* = 114), baseline characteristics are shown in Table 1. The age at PF diagnosis was highly variable with a median age of 64 years (range 31–84). Two patients had variants in two telomere related genes, one patient was homozygous for a *TERT* variant and the other 111 patients were heterozygous carriers of one variant (Fig. 1). Results for the total cohort (*n* = 177) are supplied in table E1-E7.

**Table 1:** Baseline characteristics of rare TRG-variant carrying patients with pulmonary fibrosis stratified by presence of cancer

	All (*n* = 114)	No cancer (*n* = 100)	Cancer (*n* = 14)	*p* ^*^
Male	64 (55)	56 (56)	8 (57)	0.94
Age at PF dx, y (range)^#^	63.8 (31–84)	64.5 (31–84)	60.2 (51–75)	0.39
Age at cancer dx, y (range)		*NA*	59.5 (34–75)	*NA*
Ever smoker^$^	77 (68)	67 (67)	10 (71)	0.89
Died	63 (56)	53 (53)	10 (71)	0.21
Lung transplantation	12 (11)	11 (11)	1 (7)^	1.00
Follow-up time after PF dx, y^#^	1.9 (3.2)	1.7 (2.7)	2.7 (4.6)	0.39
Survival time after PF dx, y (95%CI)^#^	3.2 (2.3–4.1)	3.2 (2.0-4.4)	3.0 (0-6.8)	0.87
Diagnoses				
· Idiopathic Pulmonary Fibrosis	92 (80)	82 (82)	10 (71)	0.47^§^
· Hypersensitivity pneumonitis	7 (6)	6 (6)	1 (7)	
· Non-specific interstitial pneumonia	3 (3)	3 (3)	0	
· Non-classifiable interstitial pneumonia	6 (5)	5 (5)	1 (7)	
· Pleuroparencymal fibroelastosis	2 (2)	2 (2)	0	
· Pneumoconiosis	2 (2)	1 (1)	1 (7)	
· Rheumatoid Interstitial Pneumonia	1 (1)	1 (1)	0	
· Cryptogenic organising pneumonia	1 (1)	0	1 (7)	
Mutant TRG^ǂ^				0.67
· TERT	63 (55)	56 (56)	7 (50)	
· Other (RTEL1, PARN, ZCCHC8, POT1, ACD, TERC)	51 (45)	44 (44)	7 (50)	
Telomere length^ǁ^				
T/S_age adjusted_	-0.248 (0.18)	-0.257 (0.19)	-0.240 (0.12)	0.56
· <10th percentile	77 (69)	66 (67)	11 (85)	0.19
· <1st percentile	45 (40)	42 (42)	3 (23)	0.18

*p for comparison between the cancer and non-cancer group. ^Lung transplantation was performed 24 years after breast cancer diagnosis (patient 13 Table 3). Values are presented as number with percentage between brackets, *n* (%), continuous values are presented as median with interquartile (IQR), unless otherwise stated. ǂTwo patients had rare variants in 2 telomere related genes (TRG): TERT + RTEL1 (*n* = 1) and TERT + TERC (*n* = 1) and were included in both the TERT and in the other group. $*n* = 112; §IPF vs. all non-IPF, Fisher exact test; ǁ*n* = 112, including 5 measurements from patients with myelodysplastic syndrome ; #*n* = 110; †including Telomere Length (TL) < 1st percentile; *Dx* diagnosis, *PF* pulmonary fibrosis, *y* years, *95%CI* 95% confidence interval, *ILD* interstitial lung disease, T/S ratioage adjusted, Telomere length adjusted for age calculated by the difference between observed T/S ratio and the age adjusted normal value

### Cancer observations

Cancer was observed in 14 of 114 TBD-PF patients, resulting in a cancer incidence of 12.3%. The expected all cancer incidence for a Dutch cohort with the same age and sex distribution is 18.9%, Table 2). Comparable results were obtained when patients with TRG variants classified as VUS were included (table E2-E4).

**Table 2:** Observed cancers in patients with pulmonary fibrosis and a P/LP TRG-variant

	Observed (*n*)	Observed cancer incidence (%)	Expected cancer incidence (%)	Observed/Expected (95% CI)
All cancers*	14	12.3	18.9	0.6 (0.4-1.0)
MDS	6	5.3	0.1	**55.5 (20.7-117.1)**
Lung	3	2.6	2.4	1.1 (0.2–3.1)
Rectum	1	0.9	0.8	1.1 (0.03–6.1)
Colon	1	0.9	1.4	0.6 (0.01–3.4)
Breast^#^	3	6.0	6.3	1.1 (0.2–2.6)

*based on the Netherlands Cancer Registry, adjusted for age and sex

^#^ percentage as part of the female population *CI* confidence interval, *MDS* myelodysplastic syndrome

bold = significant at *p* < 0.05

The median age at cancer diagnosis was 60 (range 34–75) years, telomere length and transplant-free survival was comparable between patients with and without cancer (Table 1, supplementary file fig E1A-C). In 8 patients (57%), cancer was diagnosed before the PF diagnosis (Table 3).

**Table 3:** Characteristics of patients with PF as major TBD manifestation with a P/LP TRG-variant and cancer

Patient #	Family #	Sex	Cancer type	PF dx	Age dx PF	Time (y) between PF and Ca dx	Gene	ACMG/AMP classification	History of smoking	Telomere length
1	94	M	Lung	IPF	58	1.3	*PARN*	LP	ever	< 10th
4	41	M	Lung	Pn	59	6.6	*TERT*	P	ever	< 10th
5	2	M	Lung	IPF	75	0.0	*TERT*	LP	ever	< 10th
7	26	M	Rectal	IPF	70	5.0	*RTEL1*	P	never	<1st
8	4	F	Colon	IPF	65	-1.7	*TERT*	LP	never	< 10th
12	4	F	Breast	IPF	71	-11.8	*TERT*	LP	never	> 10th
13	41	F	Breast	IPF	51	-17.0	*TERT*	P	ever	< 10th
14	24	F	Breast	COP	57	-7.5	*RTEL1*	LP	ever	> 10th
15	19	M	MDS	IPF	62	-6.3	*ZCCHC8*	LP	ever	< 10^th‡^
16	19	M	MDS	IPF	63	0.0	*ZCCHC8*	LP	ever	< 10^th‡^
17	57	M	MDS	IPF	69	-4.7	*PARN*	LP	ever	< 10^th‡^
18	19	F	MDS	IPF	52	-0.4	*ZCCHC8*	LP	ever	NA^§^
20	34	M	MDS	NCIP	58	2.1	*TERT*	P	never	<1st
21^*^	58	F	MDS	HP	55	-1.3	*TERT*	LP	ever	<1^st‡^

Ca cancer, dx diagnosis, PE pulmonary fibrosis, TRG telomere related gene, y years, Cancer types: Unk primary tumor unknown, MDS myelodysplastic syndrome, IPF idiopathic pulmonary fibrosis, Pn pneumoconiosis, COP cryptogenic organizing pneumonia, NCIP non-classifiable interstitial pneumonia, HP hypersensitivity pneumonitis, VUS variant of unknown significance, LP likely pathogenic, P pathogenic, Telomere Length (TL): <1st, TL below first percentile of controls; <10th, TL below tenth percentile of controls; >10th, TL above tenth percentile of controls. The family connection between the patients within families #4, #19 and #41 is sibling

*genetically analyzed because of clinical signs of a telomere biology disorder (TBD)

^$^no sample prior to stem cell transplantation available

^‡^measured during MDS

Sixty-three (55%) of the patients were carrier of a *TERT* variant. Cancer was observed in 7 of these patients, resulting in a cancer incidence of 11.1%. The expected all cancer incidence for a Dutch cohort with the same age and sex distribution is 7.0%, table E5).

### Myelodysplastic syndrome

Hematological malignancies were documented in 6 patients (5.0%) with all having a diagnosis of MDS. The observed number of MDS in our cohort was 56 (95% CI (21–117)) times higher than expected (Table 2). Median age at MDS diagnosis was 58 years (range 52–64) with three patients having MDS diagnosed prior to PF. MDS characteristics and specification of treatment are provided in table E6. To exclude the influence of other family-shared (epi)genetic factors on outcome, we confined the analysis to probands only: 4 out of 67 probands had MDS (6.0%), which is significantly higher than expected (O/E = 66 (95% CI: 18–161)). In *TERT* variant carriers the observed MDS incidence was 106 (95%CI (13–367)) times higher than expected (table E5).

### Solid tumors

Solid tumors (*n* = 8) were reported in eight patients (7.0%) (Table 2). From five of the eight (63%) solid tumors, a histological diagnosis was documented. Four tumors were adenocarcinoma (breast cancer *n* = 1, lung cancer *n* = 1, colon cancer *n* = 1, and rectal cancer *n* = 1) The observed cancer incidence for each solid tumor type was comparable to the expected incidence adjusted for age and sex (Table 2). Details regarding cancer treatment are provided in table E7.

### Cancer observations in TBD-cohort studies

We searched the literature for reports of cancer incidence in patients with TBD and rare TRG-variants. Cancer incidence was described for six cohorts in eleven papers, including two FPF cohorts [14, 15], one FPF-cohort consisting of both PF patients (40%) and their *TERT* variant carrying relatives [11], one TRG variant carriers (89% ILD diagnosis) cohort [27], one phenotypically diverse TBD-cohort [24, 25] and one TBD-BMF-cohort [16–18, 26] which was also part of a cohort described by Gutierrez et al. [31]. Findings are summarized in Table 4. From the five TBD-BMF-cohort papers only the most recent paper from 2024 is summarized in the table because it reports the most comprehensive overview and includes O/E ratios, making it the most suitable for direct comparison. For the phenotypically diverse TBD-cohort, both papers by Schratz et al. are summarized as the O/E details of the hematological cancers are only shown in the 2020 paper, while the 2023 paper provides a more comprehensive overview of the solid cancer spectrum.

**Table 4:** Studies reporting cancer incidence in patients with TBD

Study details, *n*, age, median (range), years	Cohort description (Diagnoses (%))	Affected gene, *n* (%)	Cancer type	*n* (%)	O/E (95%CI)	Age, (median (range)), years
Cronkhite 2008 [20], *n* = 20, 56 (37–74)	FPF patients (PF (100))	*TERT/TERC*, 20 (100)	All	5 (25)	NA	NA
			Excluding skin cancer			
Diaz de Leon 2010 [11], *n* = 134, 54 (5–88)	PF patients and relatives, 20 families (PF (40))	*TERT*, 134 (100)	All^*^	14 (10.5)	NA	NA
			Lung	3 (2.2)	NA	NA
			Breast	3 (2.2)	NA	NA
			Stomach	2 (1.5)	NA	NA
			Lymphoma	2 (1.5)	NA	NA
			Others (endometrial, skin)	4 (3.0)	NA	NA
			MDS/AA	4 (3.0)	NA	NA
Newton 2016 [15], *n* = 115, 58 (± 10)^†^	FPF 64 families (PF (100))	*TERT*, 75 (65); *TERC*, 7 (6); *RTEL1*, 14 (12); *PARN* 19, (17)	All^*^	11 (9.6)	NA	NA
			Lung	2 (1.7)	NA	NA
			Breast	4 (3.0)	NA	NA
			Lymphoma	2 (2.0)	NA	NA
			Other (oral SCC, endometrial, ovarian)	3 (3.0)	NA	NA
			MDS/AA	4 (3.5)	NA	NA
			Excluding non-melanoma skin cancers			
Schratz 2020^ǁ^ [24], *n* = 180, 50 (1–81)	Johns Hopkins Telomere Syndrome Registry (2003–2019) 113 families (TBD (100), of which classic DC (8))	*TERT*, 76 (42); *TERC*, 22 (12); *RTEL1*, 18 (10); *DKC1*,15 (8); *PARN*, 2 (1); *NAF1*, 3 (2); *TINF2*, 3 (2); *ZCCHC8*, 3 (2); Unknown, 38 (21)	All	24 (12.8)	NA	
			Solid Tumors^§^	5	**(35 (25–53))**	
			SCC tongue/oral cavity	2/2	61 (0-140)	25,35/36
			SCC anal	2	NA	33,53
			AC rectal	1	NA	38
			MDS	14	**145 (81–216)**	4 < 50; 14 ≥ 50
			AML	4	**21 (4–31)**	
			Hematological	18	NA	(53 (12–71))
Schratz 2023^ǁ^ [25], *n* = 226, 50 (0.6–81)	Johns Hopkins Telomere Syndrome Registry (2003–2022) (TBD (100))	*TERT*, 99 (44); *TERC*, 27 (12); *RTEL1*, 25 (11); *DKC1*, 17 (8); *PARN*, 8 (4); *NAF1*, 5 (2);*TINF2*, 3 (1); *ZCCHC8*, 3 (1); Unknown, 39 (17) of which 7 (3) with low TR	All	35 (15.5)^††^	NA	NA
			SCC oral cavity	7	**16.2 (5.3–30.1)**	(38 (22–60))
			SCC Anal^‡‡^	3		33, 53, 71
			SCC Esophagus	1		35
			Anorectal AC	1		38
			SCC Skin	2		52, 64
			Unknown Primary, SCC	1		58
			Lung AC (donor-derived)	1		74
			SCC all^§§^		**3.5 (1.7–5.5)**	(46 (22–71))^ǁǁ^
			MDS/AML	24 (10.6)		
			Excluding resected non-melanoma skin cancers, breast or prostate cancers			
Brudon 2024 [27], *n* = 233, 57.3 (50.1–65.4)	Bichat registry, TRG-variant carriers (ILD diagnosis (89))	NA	Lung (3 AC, 2 SCC, 1 SCLC)	6 (2.5)	NA	NA
Niewisch 2024 [18] **, *n*=230, (34.6 (1.4–82.2), at last follow-up	NCI IBMFS cohort 2002–2022 (TBD, (100)), 107 families	*DKC1*, (14.4); *TERC*, (15.2); *TERT*, (29.6); *RTEL1*, (20.4); *TINF2*, (11.7); *PARN*, (3.5); *WRAP53*, (1.3); *CTC1*, (2.6); *ACD*, 3 (1.3);	All^*^	34	**3.4 (2.3–4.7)**	42.9 (25.2–67.5)
			Solid Tumor	23	**2.6 (1.7–3.9)**	41.4 (25.2–66.4)
			HNSCC	14	**54.4 (29.7–91.2)**	43.1 (25.2–62.8)
			*Tongue*	12	**158.5 (81.9-276.9)**	43.1 (25.2–62.8)
			Digestive system††	6	**4.5 (1.6–9.7)**	37.7 (34.0-66.4)
			*Esophagus*	3	**44.2 (9.1-129.3)**	38.1 (35.4–66.4)
			*Anorectal and rectosigmoid region* ^***^	3	**10.2 (2.1–29.9)**	37.3 (34.0-38.8)
			Breast	1	0.54 (0.01-3.0)	62.7 (NA)
			Female genital system^§§§^	2	2.5 (0.3-9.0)	43.1 (36.9–49.3)
			Cervix uteri	1	5.3 (0.1–29.6)	36.9 (NA)
			All lymphatic and hematopoietic diseases^*^	11	**9.3 (4.7–16.8)**	44.2 (27.5–67.5)
			*NHL*	5	**10.8 (3.5–25.2)**	44.5 (32.2–65.6)
			*Leukemia*	6	**14.3 (5.2–31.1)**	43.7 (27.5–67.5)
			*AML*	5	**49.5 (16.1-115.5)**	44.2 (27.5–67.5)
			MDS^‡‡‡^	17	**529.7 (308.6-848.1)**	51.4 (5.9–66)
			Excluding nonmelanoma skin cancer			
Van der Vis, this study, *n* = 114, 64 (31–84)	PF patients from 113 families (PF (100))	*TERT*, 61 (54); *RTEL1*, 24 (21); *PARN*, 8 (7); *ZCCHC8*, 9 (8); *TERC*, 5 (4); *ACD*, 5 (4); *TERT + TERC*, 1 (1); *TERT + RTEL1*, 1 (1)	All	14 (12.3)	0.6 (0.4–0.9)	(59 (34–75))
			Lung	3 (2.6)	1.2 (0.4–2.7)	58, 59, 75
			Rectum	1 (0.9)	0.7 (0.01–3.8)	70
			Colon	1 (0.9)	0.4 (0.007-2.2)	65
			Breast	3 (6.0)	0.5 (0.1–1.6)	34, 50, 59
			MDS	6 (5.3)	38 (16–77)	(58 (52–64))
			Excluding skin BCC			

*AA* aplastic anemia, *MDS* myelodysplastic syndrome, *PF* pulmonary fibrosis, *DC* dyskeratosis congenita, *BCC* basal cell carcinoma, *SCC* squamous cell carcinoma, *AC* adenocarcinoma, *SCLC* small cell lung cancer, *TBD* telomere biology disorder, *NA* not available, *NHL* Non Hodgkin Lymphoma, *TRG* telomere related gene, *TR* telomerase RNA, Bold = significant at *p*<0.05; Italicized=subset of category in the row above; *MDS not included; †mean age (SD); ǁoverlapping patient cohorts, data from both papers are provided because the O/E details of the hematological cancers were only provided in the paper from 2020 and the solid cancer spectrum is more comprehensively described in the paper from 2023§one patient both SCC anal and SCC oral cavity; 4 out of 5 patients with solid cancer had a DKC1 variant and classic mucocutaneous features of dyskeratosis congenita; ‡number of individuals with cancer, some individuals where suffering from more than one cancer type. ‡‡1 patient both SCC esophageal and anorectal adenocarcinoma, 1 patient both SCC anal and SCC gingiva. §§Lung SCC was excluded from the all-SCC comparison, ǁǁcalculated from the reported data; **cancer risk prior to hematopoietic cell, lung, or liver transplantation; ††esophagus, stomach, small intestine, colon, rectum, anus; *** rectum, rectosigmoid junction, anus, anal canal and anorectum; §§§uterus, cervix, vagina, vulva, others; ‡‡‡prior to hematopoietic cell transplant, irrespective of lung or liver transplant

## Discussion

This study provides the first comparison of cancer incidence in TBD-PF patients and the general population. The all-cancer incidence of 12.3% in TBD-PF patients was comparable with the expected age- and sex-adjusted incidence of 18.9% in the general Dutch population. However, MDS occurred in 5.0% of patients, significantly exceeding the expected incidence by 56-fold.

To date, cancer incidence has been reported in only five other TBD cohorts, with 100 − 78% of patients with a known rare TRG-variant (Table 4). Three cohorts included rare TRG-variant carrying subjects of FPF families and consisted of 20 (100% PF) [14], 115 (100% PF) [15], and 134 (40% PF) [11] individuals. The all-cancer incidence in the two sufficiently powered FPF studies was 10%, aligning with our 12% (Table 4) [11, 15] Cancer types in those studies and our study overlapped almost entirely, supporting the representativeness of our cohort regarding both cancer spectrum and incidence in TBD-PF cohorts. One of the studies included only patients with *TERT* variants [11], and reported a cancer rate comparable to that observed in our study, as well as to the rate found in analyses restricted to *TERT* variant carriers within our cohort. Our study is the only TBD-PF study that compared cancer incidence with the general population, revealing an overall cancer incidence in TBD-PF patients (O/E 0.7 (95% CI 0.4-1.0).

Notably, the percentage of TBD-PF patients developing lung cancer ranges between 1.7 and 2.8% [11, 15, 27](Table 4) and is comparable to lung cancer incidence in the general population and the 2.5% lung cancer incidence in a French TRG-variant cohort (89% ILD, Table 4) [27]. In contrast, previous studies have shown that lung cancer is highly prevalent in general cohorts of patients with IPF, meta-analysis showed that the average prevalence of lung cancer is 13.7% (95%CI 10.2–17.3) [32]. Additional studies are needed to determine the respective effects of the genetic defect and the presence of PF regarding risk of lung cancer.

Upon comparison, cancer observations in non-PF based TBD cohorts are different from PF based cohorts. TBD-BMF patients had a 3–4 fold increased cancer risk compared with the general population [18, 26] and were markedly younger with a median age at cancer diagnosis of 43 years (range 25–68) in the TBD-BMF cohort compared with 60 years (range 34–75) in our TBD-PF cohort [18]. If we zoom in on the solid cancers, both the cancer spectrum and the histologic types differed (Table 4). In TBD-PF the vast majority of cancers were adenocarcinoma. In TBD-BMF the majority of cancers were squamous cancer cell (SCC) of the head/neck (HNSCC), while these cancer types were not observed in our TBD-PF population. Importantly, SCC were significantly more frequently observed in TBD-BMF than would be expected based on the general population [18, 26]. In literature, the most frequently reported solid tumors in DC patients were HNSCC (40%), skin SCCs (13%), and anorectal cancer (10%) and these patients were substantially younger at time of cancer diagnosis compared with cancer patients in the general population [16]. Moreover, in the Johns Hopkins Telomere Syndrome Registry frequency of SCC was also significantly increased 3.5 fold [25], which was largely due to the high prevalence of SCC oral cavity (Table 4). Of note, in that study the proportion of solid cancer in patients with PF as the only manifestation of TBD is not provided. However, the median age at diagnosis of SCC oral cavity was 38 years and at diagnosis of SCC-all 46 years, which is considerably lower than the age at cancer diagnosis (60 years) in our TBD-PF cohort [25]. Overall, the solid cancer spectrum differs markedly between patients with TBDs other than PF and those with TBD-PF, suggesting links between specific cancer risks and TBD phenotypes.

While findings on solid cancers differed between younger TBD-BMF and older TBD-PF-based cohorts, all studies consistently reported an increased risk of hematological cancers. Previously reported percentage of bone marrow failure (BMF) in TBD-PF cohorts were 3.0% [11] and 3.5% [15] and strikingly comparable with our results (MDS 5.0%). We are the first to provide the O/E incidence ratio and found a significant 56-fold increase of MDS in TBD-PF patients. Interestingly, the magnitude of increase in MDS was considerably larger in TBD-BMF (O/E: 530 (95%CI 309–848)) [18] and in the diverse TBD (O/E: 145 (81–216)) [24] cohorts than in our TBD-PF (O/E: 56 (21–117)) cohort. Both MDS and PF are manifestations of TBD, and co-occurrence of MDS and PF has been observed within families [11, 13, 33], as well as within patients [13, 15, 34, 35]. Alder et al. observed an overlap in age of onset in patients with solely PF (58 years, range (25–77) and patients with both BMF and PF (56 years (48–68)) [36] whereas patients with solely BMF were significantly younger (24 years (3–48)) [36]. In our cohort, the age at onset of MDS was 58 years (range 52–64), which is almost two decades earlier than the median age of onset of sporadic MDS in the general Dutch population [37]. Hence, patients with TBD-PF are at high risk of MDS, warranting surveillance during follow-up.

This study has several limitations. First, its retrospective nature may introduce bias. Although disease history is always inquired, it may have been incomplete or may have influenced the decision to perform genetic analysis. Furthermore, data on the cause of death were incomplete. Therefore, we were not able to perform analyses accounting for competing risks of death due to pulmonary fibrosis, future studies will be essential to better characterize this. Additionally, distinguishing MDS from BMF in TBD patients with cytopenias can be challenging. Observations of MDS were based on medical reports from hematologists and referring pulmonologists, but not in all cases cytogenetic and molecular abnormalities were present, potentially leading to over reporting MDS. However, hematological surveillance is essential not only for patients at risk of MDS, but equally for those at risk of BMF. Second, cancer incidence in relatives was not studied. While current findings are applicable for PF patients wherein a rare TRG-variant is detected, they may also apply to (asymptomatic) rare TRG-variant carrying siblings in the same age-range. In offspring, however, anticipation may occur which may influence cancer risk. This study lays the groundwork for further evaluation of differences in cancer incidence across multiple generations to elucidate cancer risk in consecutive generations of families with rare TRG variants.

## Conclusions

Overall, our findings demonstrate that individuals with TBD-PF have an all-cancer risk comparable to that of the general population. However, TBD-PF patients are at increased risk of developing MDS, emphasizing the importance of tailored genetic counselling and hematological surveillance.

## Supplementary Information

Supplementary Material 1.

## Data Availability

The dataset used and analyzed during the current study are available from the corresponding author on reasonable request.

## Abbreviations

AA: Aplastic Anemia
ACMG: American College of Medical Genetics and Genomics
AML: Acute Myeloid Leukemia
BMF: Bone Marrow Failure
CI: Confidence Interval
CuI: Cumulative Incidence
DC: Dyskeratosis Congenita
Dx: Diagnosis
FPF: Familial Pulmonary Fibrosis
HNSCC: Squamous Cell Carcinoma of the Head and Neck
ILD: Interstitial Lung Disease
IPF: Idiopathic Pulmonary Fibrosis
LP: Likely Pathogenic
MDS: Myelodysplastic Syndrome
NCR: Netherlands Cancer Registry
NHL: Non Hodgkin Lymphoma
O/E: Observed/Expected
P: Pathogenic
PF: Pulmonary Fibrosis
SCC: Squamous Cell Carcinoma
SD: Standard Deviation
TBD: Telomere Biology Disorders
TBD-PF: rare telomere related gene variant carrying patients with Pulmonary Fibrosis as major manifestation of Telomere Biology Disorders
TRG: Telomere Related Gene
VUS: Variant of Unknown Significance
