# Supplementary material for: Cancer risk in patients with pulmonary fibrosis and a rare telomere related gene variant
Source: Respir Res. 2026 Jan 19;27:61. doi: 10.1186/s12931-025-03469-2 (PMC12896182; doi:10.1186/s12931-025-03469-2)
Supplement: Supplementary file 1 — Supplementary Material 1. [file 12931_2025_3469_MOESM1_ESM.docx]

**Supplement to “Cancer risk in patients with pulmonary fibrosis and a rare telomere related gene variant”**

Joanne J. van der Vis^1,2*^, Martijn T. K. Maus, Charlotte I. de Bie, Jasper J. van der Smagt, Laura G. M. Daenen, Matthijs F.M. van Oosterhout, Jan C. Grutters and Coline H. M. van Moorsel

^1.^ ILD Center of Excellence, member of European Reference Network-Lung, St Antonius Hospital, Nieuwegein, the Netherlands

^2.^ Department of Clinical Chemistry, St. Antonius Hospital, Nieuwegein, The Netherlands

*Corresponding author:

E-mail: a.vandervis@antoniusziekenhuis.nl

**Supplementary Methods**

**Telomere length**

Telomere length was measured in DNA from total peripheral blood by quantitative PCR (qPCR) by determining T/S ratio as described previously (1,2). As a reference cohort T/S ratios for 164 healthy individuals with an age ranging between 20 and 70 years of age was determined. Age-adjusted normal values for the T/S ratio were calculated by determining the best-fitting linear regression line through the healthy control data, and percentiles were derived from the regression line. Telomere length adjusted for age (T/S_age adjusted_) was calculated by the difference between observed T/S ratio and the age adjusted normal value. Since telomere length was measured in total leukocytes, including myeloid-derived cells, the elevated cell turnover seen in myelodysplastic syndrome could have influenced the observed telomere shortening.

**Genetic analysis**

In all patients genetic analysis for germline rare telomere related gene variants was performed in DNA isolated from total peripheral blood. Patients diagnosed prior to 2018 were initially screened for rare variants in *TERT* and *TERC* by Sanger sequencing (n=25).If no rare variant was identified in these genes, whole exome sequencing (WES) was subsequently performed (n=102). WES data was analyzed for single nucleotide variants and small insertions and deletions in a panel consisting of telomere related genes associated with adult FPF, including *ACD, DKC1, NAF1, , PARN, POT1, RTEL1, TERC, TERT, TINF2,* and *ZCCHC8*. When a disease-causing variant was identified in the proband of a family, cascade testing was performed in affected relatives, targeting only the identified familial variant (n=50). All genetic analyses were performed in DNA isolated from total peripheral blood.

Results of the cytological, cytogenetic, and molecular classification of MDS were obtained from laboratory reports included in the patient charts (table E5).

**Cancer types**

The Netherlands Cancer Register (NCR) registered cancer types include invasive cancer types, and the non-invasive cancer types ductal carcinoma in situ (DCIS) and upper tract urothelial carcinoma. In our TBD-PF cohort basal cell carcinoma (BCC) was noted 9 times, yet excluded from analysis due to well-known under registration and absence of BCC in the NCR

**Statistical analysis**

Study data were collected and managed using research redcap electronic data capture (REDCap, VanderBilt University, Nashville, TN) software hosted at our institute. The statistical analysis was performed using SPSS29 (IBM Corp., Armonk, NY) and GraphPad Prismv.8 (Graphpad software INC., San Diego, CA). To analyze differences between groups, Pearson Chi square test, Fisher exact test, Mann-Whitney U-test and independent t-test were used, as appropriate. The Kaplan-Meier method with log-rank test was used to analyze transplant-free survival time after pulmonary fibrosis diagnosis, both lung transplantation and death were considered as events. Differences with a p-value <0.05 were considered statistically significant.

**Supplementary results**


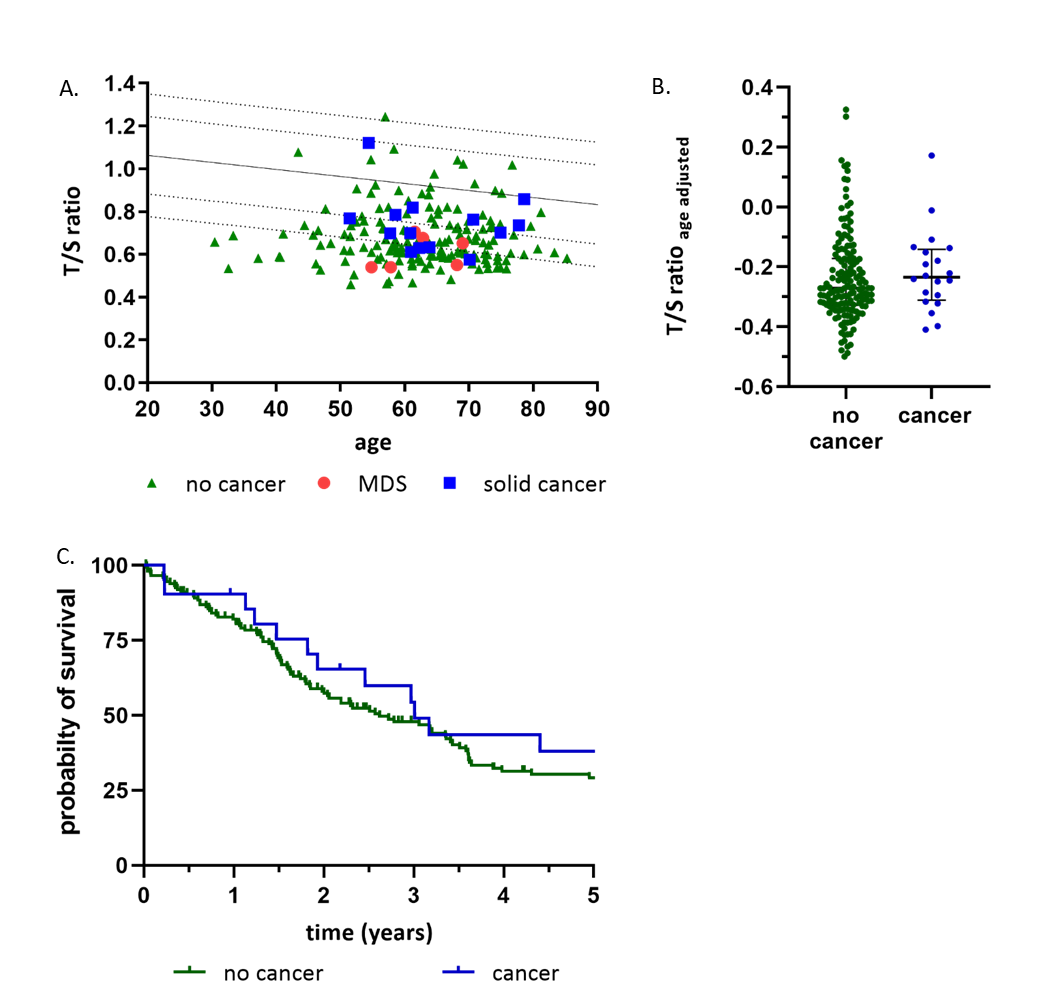


**Fig E1 Telomere length in total leukocytes in peripheral blood and transplant-free survival in rare TRG-variant carrying patients with PF as major telomere biology disease manifestation (TBD-PF) stratified by cancer presence**. A. Telomere length of TBD-PF patients without cancer (n=155, green triangles), with myelodysplastic syndrome (MDS, n=6 (of which 5 were measured during MDS, red dots), and with solid cancer (n=14, blue rectangles) measured by quantitative PCR assay (T/S ratio) plotted against age. The solid line indicates the 50^th^ percentile of control subjects; the lower dotted lines indicate the 1^st^ and 10^th^ percentile of control subjects. B. Telomere length adjusted for age calculated by the difference between observed T/S ratio and the age adjusted normal value (T/S_age adjusted_) for TBD-PF patients without cancer (n=155) versus with cancer (solid cancer n=14; MDS n=6)) (-0.272 vs -0.235, p=0.22). C. Kaplan-Meier survival curve showing a median transplant-free survival of TBD-PF patients without cancer of 2.6 years (95%CI 1.8-3.5; n=152, green line) versus TBD-PF patients with cancer of 3.0 years (95%CI 2.0-4.0; n=21, blue line), p=0.9, vertical bars indicate censored

**Table E1.** Rare variants in telomere related genes in patients with pulmonary fibrosis included in this study

| Gene | Mutation | Transcript | Effect on protein | ACMG classification | Number of families | Number of patients | References |
| --- | --- | --- | --- | --- | --- | --- | --- |
| *ACD* | c.46C>A | NM_001082486.2 | p.(Leu16Arg) | VUS | 1 | 1 |  |
| *ACD* | c.215C>A | NM_001082486.1 | p.(Ala72Glu) | VUS | 1 | 1 | (3) |
| *ACD* | c.250_252del | NM_001082486.2 | p.(Lys84del) | LP | 1 | 2 | (3–5) |
| *ACD* | c.250A>G | NM_001082486.2 | p.(Lys84Glu) | LP | 2 | 3 | (3) |
| *PARN* | c.98C>T | NM_001134477.2 | p.(Pro33Leu) | LP | 3 | 5 | (6–9) |
| *PARN* | c.224A>T | NM_001134477.2 | p.(Gln75Leu) | VUS | 1 | 1 |  |
| *PARN* | c.245+5G>A | NM_002582.4 | p.(Lys59fs)^$^ | P | 1 | 1 |  |
| *PARN* | c.347A>G | NM_001134477.2 | p.(Gln116Arg) | VUS | 1 | 1 |  |
| *PARN* | c.862C>T | NM_001134477.2 | p.(Arg288Trp) | VUS | 1 | 1 | (10) |
| *PARN* | c.982T>C | NM_001134477.2 | p.(Cys328Arg) | VUS | 1 | 1 |  |
| *PARN* | c.1214G > C | NM_001134477.2 | p.(Ser405Thr) | VUS | 1 | 1 | (7,8) |
| *PARN* | g.14656226_14715540del c.1214G>C^‡^ | NC_000016.9 NM_001134477.2 | exon 6-18del p.(Ser405Thr) | LP VUS | 1 | 2 |  |
| *POT1* | c.776T>C | NM_015450.2 | p.(Leu259Ser) | VUS | 1 | 4 | (11) |
| *POT1* | c.1071dup | NM_015450.2 | p.(Gln358Serfs) | VUS | 1 | 1 | (12) |
| *RTEL1* | c.62A>T | NM_001283009.2 | p.(Gln21Leu) | VUS | 1 | 1 |  |
| *RTEL1* | c.1037G>A | NM_001283009.2 | p.(Ser346Asn) | LP | 2 | 2 |  |
| *RTEL1* | c.1038-5G>A | NM_001283009.2 | p.(?) | VUS | 1 | 1 |  |
| *RTEL1* | c.1073C>T | NM_001283009.2 | p.(Tyr358Met) | VUS | 1 | 1 |  |
| *RTEL1* | c.1691C>T | NM_001283009.2 | p.(Pro564Leu) | VUS | 1 | 1 |  |
| *RTEL1* | c.1972_1974del | NM_001283009.2 | p.(Leu658del) | LP | 2 | 5^#^ | (7,8) |
| *RTEL1* | c.2035C>G | NM_032957.5 | p.(Arg679Gly) | LP | 2 | 4 | (13) |
| *RTEL1* | c.2186 G>A | NM_001283009.2 | p.(Arg729His) | VUS | 3 | 3 | (14) |
| *RTEL1* | c.2870G>A | NM_001283009.2 | p.(Arg957Gln) | LP | 1 | 1 | (15) |
| *RTEL1* | c.2890del | NM_032957.5 | p.(Ala964fs) | LP | 1 | 1 |  |
| *RTEL1* | c.2892T>G c.3790C>T^‡^ | NM_001283009.2 | p.(Phe964Leu)  p.(Arg1264Cys) | LP | 1 | 1 | (16) |
| *RTEL1* | c.2956C>T | NM_001283009.2 | p.(Arg986 *) | P | 2 | 4 | (7,14,17–23) |
| *RTEL1* | c.3002C>T | NM_001283009.2 | p.(Ala1001Val) | VUS | 1 | 1 |  |
| *RTEL1* | c.3343_3345del | NM_001283009.2 | p.(Asp1115del) | VUS | 1 | 1 |  |
| *RTEL1* | c.3343+15C>T | NM_001283009.2 | p.(?) | VUS | 1 | 1 |  |
| *RTEL1* | c.3392C>A | NM_001283009.2 | p.(Thr1131Lys) | VUS | 1 | 1 |  |
| *RTEL1* | c.3730_3731del | NM_032957.5 | p.(Cys1244fs) | VUS | 1 | 1 | (24) |
| *RTEL1* | c.3757G>C | NM_001283009.2 | p.(Val1253Leu) | VUS | 1 | 2 |  |
| *RTEL1* | c.3791G>A | NM_001283009.2 | p.(Arg1264His) | LP | 1 | 2 | (17,23,25–29) |
| *RTEL1* | c.3820C>T | NM_001283009.2 | p.(Gln1274Ter) | LP | 1 | 5 |  |
| *RTEL1* | c.3811C>T | NM_001283009.2 | p.(Arg1271Trp) | VUS | 1 | 2 | (8) |
| *TERC* | r.30G>A | NR_001566 |  | VUS | 1 | 1 | (7) |
| *TERC* | r.54-57delAACU | NR_001566 |  | LP | 1 | 1^#^ | (23,30,31) |
| *TERC* | r.91G>C | NR_001566 |  | VUS | 1 | 1 | (7) |
| *TERC* | r.216-229del14 | NR_001566 |  | P | 1 | 1 | (32) |
| *TERC* | r.304_305delAG | NR_001566 |  | P | 1 | 2 |  |
| *TERC* | r.448A>G | NR_001566 |  | LP | 1 | 2 | (7,26) |
| *TERT* | c.89G>T | NM_198253.3 | p.(Arg30Leu) | VUS | 1 | 1 |  |
| *TERT* | c.92T>C | NM_198253.3 | p.(Leu31Pro) | VUS | 1 | 1 |  |
| *TERT* | c.232A>T | NM_198253.3 | p.(Lys78X) | LP | 1 | 2 | (7) |
| *TERT* | c.286G>T | NM_198253.3 | p.(Val96Leu) | VUS | 1 | 2 |  |
| *TERT* | c.299G > A | NM_198253.3 | p.(Gly100Asp) | LP | 1 | 2 | (7) |
| *TERT* | c.373A>C | NM_198253.3 | p.(Asn125His) | LP | 1 | 1 |  |
| *TERT* | c.375C>G | NM_198253.3 | p.(Asn125Lys) | LP | 2 | 5 | (33) |
| *TERT* | c.395G>A | NM_198253.3 | p.(Arg132Gln) | VUS | 1 | 1 | (7,34) |
| *TERT* | c.436G>A | NM_198253.3 | p.(Asp146Asn) | VUS | 1 | 1 |  |
| *TERT* | c.455T>A | NM_198253.3 | p.(Leu152Gln) | VUS | 1 | 1 | (35) |
| *TERT* | c.515G>A | NM_198253.3 | p.(Gly172Glu) | VUS | 1 | 1 | (7) |
| *TERT* | c.973T>G | NM_198253.3 | p.(Tyr325Asp) | VUS | 1 | 1 | (33) |
| *TERT* | c.1151A>T | NM_198253.3 | p.(Gln384Leu) | VUS | 1 | 2 |  |
| *TERT* | c.1212del | NM_198253.3 | p.(Tyr405fs) | LP | 1 | 1 | (7) |
| *TERT* | c.1272dup | NM_198253.3 | p.(Val425fs) | P | 1 | 1 | (36) |
| *TERT* | c.1445del | NM_198253.3 | p.(His482ProfsX27) | P | 1 | 1 |  |
| *TERT* | c.1584T>G | NM_198253.3 | p.(Cys528Trp) | LP | 4 | 4 | (7,35) |
| *TERT* | c.1698_1700delCAC | NM_198253.3 | p.(Tyr567del) | VUS | 1 | 4 | (7,35) |
| *TERT* | c.1726C > T | NM_198253.3 | p.(Tyr576His) | VUS | 1 | 2 | (8) |
| *TERT* | c.1729C>T | NM_198253.3 | p.(Arg577Trp) | LP | 1 | 6 | (7,33) |
| *TERT* | c.1885G>A | NM_198253.3 | p.(Gly629Arg) | VUS | 1 | 1 | (20) |
| *TERT* | c.2005C>T | NM_198253.3 | p.(Arg669Trp) | P | 12 | 20* | (7,13,22,31,37) |
| *TERT* | c.2011C>T | NM_198253.3 | p.(Arg671Trp) | LP | 1 | 1 | (7,16,35,37–40) |
| *TERT* | c.2032G>A | NM_198253.3 | p.(Ala678Thr) | LP | 1 | 2 |  |
| *TERT* | c.2146G>A | NM_198253.3 | p.(Ala716Thr) | P | 1 | 1 | (7,13,15,34,35,41–43) |
| *TERT* | c.2303A>T | NM_198253.3 | p.(Asp768Val) | LP | 1 | 4 | (35) |
| *TERT* | c.2312C > T | NM_198253.3 | p.(Pro771Leu) | LP | 3 | 7 |  |
| *TERT* | c.2317A>G | NM_198253.3 | p.(Met773Val) | VUS | 1 | 1 |  |
| *TERT* | c.2321G>A | NM_198253.3 | p.(Arg774Gln) | LP | 1 | 1 | (22) |
| *TERT* | c.2377G>A | NM_198253.3 | p.(Glu793Lys) | VUS | 1 | 1 | (7,16) |
| *TERT* | c.2406C>G | NM_198253.3 | p.(Ser802Arg) | VUS | 1 | 1 | (35) |
| *TERT* | c.2456G>A | NM_198253.3 | p.(Arg819His) | VUS | 1 | 1 | (7) |
| *TERT* | c.2603A>G | NM_198253.3 | p.(Asp868Gly) | LP | 1 | 1 | (23,33,44) |
| *TERT* | c.2798T>C | NM_198253.3 | p.(Leu933Pro) | VUS | 1 | 1 |  |
| *TERT* | c.2884C>T | NM_198253.3 | p.(Arg962Cys) | VUS | 1 | 1 |  |
| *TERT* | c.2990T>G | NM_198253.3 | p.(Val997Gly) | VUS | 1 | 1 |  |
| *TERT* | c.3072_3073delinsT | NM_198253.3 | p.(Gln1024HisfsX24) | P | 1 | 1 |  |
| *TERT* | c.3101G>A | NM_198253.3 | p.(Arg1034His) | VUS | 1 | 2 |  |
| *TERT* | c.3148A>G | NM_198253.3 | p.(Lys1050Glu) | P | 1 | 1 | (7,37,38,45) |
| *TERT* | c.3167T>G | NM_198253.3 | p.(Leu1056Arg) | VUS | 1 | 1 | (22) |
| *TERT* | c.3187G>A | NM_198253.3 | p.(Gly1063Ser) | LP | 1 | 1 | (13,37,38) |
| *TERT* | c.3208G>A | NM_198253.3 | p.(Val1070Met) | VUS | 1 | 1 |  |
| *TERT* | c.3256C>T | NM_198253.3 | p.(Arg1086Cys) | VUS | 1 | 4 | (20,36) |
| *ZCCHC8* | c.557C>T | NM_017612.5 | p.(Pro186Leu) | LP | 1 | 1 | (46–48) |
| *ZCCHC8* | c.586G>A | NM_017612.5 | p.(Glu196Lys) | LP | 2 | 8 | (47) |

^#^1 patient also heterozygous for *TERT* c.2005C>T mutation; *1 patient homozygous; ^$^ confirmed with RNA analysis; ^ǂ^ allelic phase unknown; VUS, variant of unknown significance; LP, likely pathogenic; P, pathogenic

**Table E2. Baseline characteristics of patients with pulmonary fibrosis and a rare TRG variant classified as variant of unknown significance, likely pathogenic or pathogenic stratified by presence of cancer**

|  | **All (n=177)** | **No cancer (n=156)** | **Cancer (n=21)** | **p^*^** |
| --- | --- | --- | --- | --- |
| Male | 112 (63) | 99 (64) | 13 (62) | 0.89 |
| Age at PF dx, y (range)^#^ | 62.8 (30-85) | 63.2 (30-85) | 61.5 (51-80) | 0.95 |
| Age at cancer dx, y (range) |  | *NA* | 60.4 (34-81) | *NA* |
| Ever smoker^$^ | 120 (68) | 104 (67) | 16 (76) | 0.40 |
| Died | 101 (64) | 85 (63) | 16 (80) | 0.14 |
| Lung transplantation | 21 (12) | 20 (13) | 1 (5)^ | 0.48 |
| Follow-up time after PF dx, y^#^ | 1.9 (2.6) | 1.8 (2.6) | 3.0 (4.0) | 0.14 |
| Survival time after PF dx, y (95%CI)^#^ | 2.8 (2.0-3.5) | 2.6 (1.8-3.5) | 3.0 (2.0-4.0) | 0.92 |
| Diagnoses |  |  |  | 0.78^ǁ^ |
| ·  Idiopathic Pulmonary Fibrosis | 140 (79) | 124 (79) | 16 (75) |  |
| ·  Hypersensitivity pneumonitis | 9 (5) | 8 (5) | 1 (5) |  |
| ·  Non-specific interstitial pneumonia | 8 (5) | 8 (5) | 0 |  |
| ·  Non-classifiable interstitial pneumonia | 7 (4) | 7 (4) | 1 (5) |  |
| ·  Pleuroparencymal fibroelastosis | 3 (2) | 3 (2) | 0 |  |
| ·  Smoking-related ILD | 2 (1) | 2 (1) | 0 |  |
| ·  Pneumoconiosis | 2 (1) | 1 (1) | 1 (5) |  |
| ·  Reumatoid Interstitial Pneumonia | 2 (1) | 2 (1) | 0 |  |
| ·  Unclassified connective tissue disease | 1 (1) | 0 | 1 (5) |  |
| ·  Cryptogenic organising pneumonia | 1 (1) | 0 | 1 (5) |  |
| Mutant TRG^ǂ^ |  |  |  | 0.92 |
| • TERT | 96 (54) | 85 (54) | 11 (52) |  |
| • Other (RTEL1, PARN, ZCCHC8, POT1, ACD, TERC) | 83 (46) | 73 (46) | 10 (48) |  |
| ACMG classification^ǂ^ |  |  |  | 0.81 |
| • Pathogenic or likely pathogenic | 116 (63) | 102 (62) | 14 (67) |  |
| • Variant of unknown significance | 63 (38) | 56 (38) | 7 (33) |  |
| Telomere length^$^ |  |  |  |  |
| T/S_age adjusted_ | -0.271 (0.16) | -0.272 (0.16) | -0.235 (0.17) | 0.22 |
| • <10th percentile | 127 (72) | 113 (73) | 14 (70) | 0.78 |
| • <1st percentile | 78 (44) | 72 (47) | 6 (30) | 0.16 |

^*^p for comparison between the cancer and non-cancer group. ^Lung transplantation was performed 24 years after breast cancer diagnosis (patient 13 table 3). Values are presented as number with percentage between brackets, n(%), continuous values are presented as median with interquartile (IQR), unless otherwise stated ^ǁ^IPF vs all non-IPF. ^ǂ^Two patients had rare variants in 2 telomere related genes (TRG): *TERT*+*RTEL1* (n=1) and *TERT*+*TERC* (n=1) and were included in both the *TERT* and in the other group . ^$^n=175, including 5 measurements from patients with MDS; ^ǂ^including Telomere Length (TL)<1st percentile; Dx, diagnosis; PF, pulmonary fibrosis; y, years; 95%CI, 95% confidence interval; ILD, interstitial lung disease; T/S ratio_age adjusted_, Telomere length adjusted for age calculated by the difference between observed T/S ratio and the age adjusted normal value.

**Table E3. Observed cancers in patients with pulmonary fibrosis and a rare TRG variant classified as variant of unknown significance, likely pathogenic or pathogenic**

|  | **Observed (n)** | **Observed cancer incidence (%)** | **Expected cancer incidence (%)** | **Observed/Expected (95% CI)** |
| --- | --- | --- | --- | --- |
| All cancers* | 21 | 11.9 | 18.9 | **0.6 (0.4-0.9)** |
| MDS | 7 | 4 | 0.1 | **38 (16-77)** |
| Lung | 5 | 2.8 | 2.4 | 1.2 (0.4-2.7) |
| Rectum | 1 | 0.6 | 0.8 | 0.7 (0.01-3.8) |
| Colon | 1 | 0.6 | 1.4 | 0.4 (0.007-2.2) |
| Breast^#^ | 3 | 4.6 | 8.1 | 0.5 (0.1-1.6) |
| Prostate^$^ | 1 | 0.9 | 4 | 0.2 (0.005-1.2) |
| Oesophagus | 1 | 0.6 | 0.5 | 1.1 (0.02-6.4) |
| Lymphoma^¥^ | 1 | 0.6 | 0.3 | 1.7 (0.03-9.3) |
| Unknown primary | 1 | 0.6 | 0.1 | 3.9 (0.07-21.7) |

**Table E4. Characteristics of patients with PF as major TBD manifestation with a rare TRG-variant and cancer**

| **Patient #** | **Family #** | **Sex** | **Cancer type** | **PF dx** | **Age dx PF** | **Time (y) between PF and Ca dx** | **Gene** | **ACMG/AMP classification** | **History of smoking** | **Telomere length** |
| --- | --- | --- | --- | --- | --- | --- | --- | --- | --- | --- |
| 1 | 94 | M | Lung | IPF | 58 | 1.3 | *PARN* | LP | ever | <10^th^ |
| 2 | 68 | F | Lung | IPF | 61 | 0.0 | *RTEL1* | VUS | ever | <1^st^ |
| 3 | 53 | F | Lung | UCTD | 62 | -1 | *RTEL1* | VUS | ever | <1^st^ |
| 4 | 41 | M | Lung | Pn | 59 | 6.6 | *TERT* | P | ever | <10^th^ |
| 5 | 2 | M | Lung | IPF | 75 | 0.0 | *TERT* | LP | ever | <10^th^ |
| 6 | 9 | M | Unk | IPF | 58 | 1.2 | *TERT* | VUS | ever | >10^th^ |
| 7 | 26 | M | Rectal | IPF | 70 | 5.0 | *RTEL1* | P | never | <1^st^ |
| 8 | 4 | F | Colon | IPF | 65 | -1.7 | *TERT* | LP | never | <10^th^ |
| 9 | 83 | M | Lymp | IPF | 61 | -0.9 | *PARN* | VUS | never | >10^th^ |
| 10 | 76 | M | Eso | IPF | 78 | 2.9 | *TERT* | VUS | ever | >10^th^ |
| 11 | 52 | M | Prostate | IPF | 80 | -6.9 | *TERT* | VUS | ever | >10^th^ |
| 12 | 4 | F | Breast | IPF | 71 | -11.8 | *TERT* | LP | never | >10^th^ |
| 13 | 41 | F | Breast | IPF | 51 | -17.0 | *TERT* | P | ever | <10^th^ |
| 14 | 24 | F | Breast | COP | 57 | -7.5 | *RTEL1* | LP | ever | >10^th^ |
| 15 | 19 | M | MDS | IPF | 62 | -6.3 | *ZCCHC8^ǁ^* | LP | ever | <10^th‡^ |
| 16 | 19 | M | MDS | IPF | 63 | 0.0 | *ZCCHC8^ǁ^* | LP | ever | <10^th‡^ |
| 17 | 57 | M | MDS | IPF | 69 | -4.7 | *PARN^ǁ^* | LP | ever | <10^th‡^ |
| 18 | 19 | F | MDS | IPF | 52 | -0.4 | *ZCCHC8^ǁ^* | LP | ever | NA^§^ |
| 19 | 101 | M | MDS | IPF | 68 | -13.2 | *TERT*^†^ | VUS | ever | <1^st‡^ |
| 20 | 34 | M | MDS | NCIP | 58 | 2.1 | *TERT* | P | never | <1^st^ |
| 21^*^ | 58 | F | MDS | HP | 55 | -1.3 | *TERT^ǁ^* | LP | ever | <1^st‡^ |

* genetically analyzed because of clinical signs of a telomere biology disorder (TBD), Ca: cancer, dx: diagnosis, PF: pulmonary fibrosis, TRG: telomere related gene, y: years, Cancer types: Unk: primary tumor unknown, Lymp: lymphoma, Eso: Esophagus, MDS: myelodysplastic syndrome, IPF: idiopathic pulmonary fibrosis; UCTD: undefined connective tissue disease; Pn: pneumoconiosis; COP, cryptogenic organizing pneumonia; NCIP, non-classifiable interstitial pneumonia; HP, hypersensitivity pneumonitis; VUS, variant of unknown significance, LP, likely pathogenic, P, pathogenic, Telomere Length (TL): <1^st^, TL below first percentile of controls; <10^th^, TL below tenth percentile of controls; >10^th^, TL above tenth percentile of controls. The family connection between the patients within families #4, #19 and #41 is sibling. ^$^no sample for TL measurement prior to stem cell transplantation available; ^‡^measured during MDS. ^ǁ^measured during MDS, TRG variant segregated with pulmonary fibrosis in relatives who did not have a hematologic malignancy. ^†^measured during MDS, no segregation analysis possible due to unavailability of DNA from affected relatives.

**Table E5. Observed cancers in 63 patients with pulmonary fibrosis and a P/LP *TERT* variant**

|  | Observed (n) | Observed cancer incidence (%) | Expected cancer incidence (%) | Observed/Expected (95% CI) |
| --- | --- | --- | --- | --- |
| All cancers* | 7 | 11.1 | 7.0 | 11 (0.7-3.1) |
| MDS | 2 | 3.2 | 0.03 | **106 (13-367)** |
| Lung | 2 | 3.2 | 0.9 | 3.6 (0.4-12.4) |
| Colon | 1 | 1.6 | 0.5 | 3.1 (0.1-16.4) |
| Breast^#^ | 2 | 3.2 | 3.7 | 0.9 (0.2-6.1) |
|  |  |  |  |  |

*based on the Netherlands Cancer Registry, adjusted for age and sex; ^#^ percentage as part of the female population CI, confidence interval; MDS, myelodysplastic syndrome; **bold**=significant at p<0.05

**Table E6.** Details regarding myelodysplastic syndrome diagnoses and treatment

| patient nr ^*)^ | age at PF diagnosis | age at MDS diagnosis (year) | MDS characteristics | IPSS score | treatment | cytological, cytogenetic, molecular classification |
| --- | --- | --- | --- | --- | --- | --- |
| 15 | 62 | 55 (2009) | hypoplastic bone marrow with minor dysplastic changes of megakaryopoiesis; pancytopenia and red blood cell macrocytosis | 0 | watchful waiting | no cytogenetic abnormalities |
| 16 | 63 | 63 (2015) | refractory cytopenia with multilineage dysplasia | 3^#)^ | no treatment options due to severe pulmonary fibrosis | bone marrow: 46,XY[21].ish dup(11)(p15.4p15.4)(5'NUP98++).arr[hg19]11p15.4(3,737,639-4,020,840)x3, 11q23.3(118,337,573-118,355,642)x3, in skin biopsy not present |
| 17 | 69 | 64 (2013) | thrombocytopenia, anemia and red blood cell macrocytosis | NA | watchful waiting | NA |
| 18 | 52 | 52 (2015) | refractory anemia with excess blasts-2 | >4.5 | Idarubicine/Ara-C; allogenic stem cell transplant | Karyotype bone marrow: 46,XX[20].arr[hg19] 11q23.3(118328003-118353482)x3, in skin biopsy not present |
| 19 | 68 | 55 (2008) | red blood cell macrocytosis and anemia | NA | watchful waiting | NA |
| 20 | 58 | 60 (2018) | multilineage dysplasia | 1.5^$)^ | allogenic stem cell transplantation | del 2q, del 5q, del 7p, del 13q + 16 and aUPD 17p13 (pm p53 gene) |
| 21 | 55 | 53 (2015) | hypoplastic bone marrow; thrombocytopenia, anemia and red blood cell macrocytosis | 3^#)^ | no treatment due to co-morbidities | at diagnosis:  46, XX,+1,dic(1;15)(p11;p11)[6]/46,XX[14];  after 3 years: 46,XX,+1,dic(1;15)(p11;p11)[1]/46,XX,+1,dic(1;14)(p11;q3?2)[4]/46,XX[16] |

^#)^ IPSS-R *) corresponds with patient numbering in table E2; PF, pulmonary fibrosis; MDS, myelodysplastic syndrome; IPSS, international prognostic scoring system; NA, not available

**Table E7.** Details regarding treatment of solid cancers

| Patient nr*) | Age at PF diagnosis | Age at cancer diagnosis | Type of cancer | Treatment | Remarks |
| --- | --- | --- | --- | --- | --- |
| 1 | 58 | 59 | Lung | No treatment | Died shortly after cancer diagnosis |
| 2 | 61 | 61 | Lung | Unknown | Treated in other hospital; self-reported: no indication for radiotherapy |
| 3 | 62 | 60 | Lung | Stereotactic radiotherapy | Right upper lobe: T1bN0M0 |
| 4 | 59 | 65 | Lung | No treatment | Left lower lobe, no treatment due to severe pulmonary fibrosis; Died shortly after cancer diagnosis |
| 5 | 75 | 75 | Lung | Lobectomy | Left upper lobe, adenocarcinoma |
| 6 | 59 | 60 | Unknown^ǂ)^ | No treatment | Died shortly after cancer diagnosis |
| 7 | 70 | 75 | Rectal | Chemotherapy (Capecitabine) and radiotherapy | No resection due to risk for general anesthesia in context of pulmonary fibrosis; Uncomplicated chemo radiation therapy with a good response, chemotherapy induced thrombocytopenia |
| 8 | 65 | 63 | Colon | Laparoscopic sigmoid resection and chemotherapy (Capecitabine) | Cured of colon cancer |
| 9 | 61 | 60 | Lymphoma | Local radiotherapy | Stadium IV in right orbital, international prognostic index score:1 (low risk) |
| 10 | 78 | 81 | Esophagus | No treatment | Died shortly after cancer diagnosis |
| 11 | 80 | 73 | Prostate | Brachytherapy | Cured of prostate cancer |
| 12 | 71 | 59 | Breast | Radical resection and adjuvant radiotherapy of glandular disc in the context of breast-conserving surgery | Cured of breast cancer |
| 13 | 51 | 34 | Breast | Breast amputation and adjuvant radiotherapy | Cured of breast cancer |
| 14 | 57 | 50 | Breast | Breast-conserving surgery and radiotherapy | Cured of breast cancer |

*) corresponds with patient numbering in table E2; ^ǂ)^ The patient presented with a swelling of a lymph node in the left inguinal region. Biopsy showed squamous cell carcinoma, but no anatomical primary site was identified. PF, pulmonary fibrosis;

**References**

1. Snetselaar R, Van Moorsel CHM, Kazemier KM, Van Der Vis JJ, Zanen P, Van Oosterhout MFM, et al. Telomere length in interstitial lung diseases. Chest. 2015;148(4):1011–8.

2. Cawthon RM. Telomere length measurement by a novel monochrome multiplex quantitative PCR method. Nucleic Acids Res. 2009;37(3):1–7.

3. Hoffman TW, Van Der Vis JJ, Van Der Smagt JJ, Massink MPG, Grutters JC, Van Moorsel CHM. Pulmonary fibrosis linked to variants in the ACD gene, encoding the telomere protein TPP1. Eur Respir J. 2019;54(6).

4. Guo Y, Kartawinata M, Li J, Pickett HA, Teo J, Kilo T, et al. Inherited bone marrow failure associated with germline mutation of ACD, the gene encoding telomere protein TPP1. Blood. 2014 Oct 30;124(18):2767.

5. Kocak H, Ballew BJ, Bisht K, Eggebeen R, Hicks BD, Suman S, et al. Hoyeraal-Hreidarsson syndrome caused by a germline mutation in the TEL patch of the telomere protein TPP1. Genes Dev. 2014 Oct 1;28(19):2090.

6. Dressen A, Abbas AR, Cabanski C, Reeder J, Ramalingam TR, Neighbors M, et al. Analysis of protein-altering variants in telomerase genes and their association with MUC5B common variant status in patients with idiopathic pulmonary fibrosis: a candidate gene sequencing study. Lancet Respir Med. 2018;6(8):603–14.

7. Justet A, Klay D, Porcher R, Cottin V, Ahmad K, Molina MM, et al. Safety and efficacy of pirfenidone and nintedanib in patients with idiopathic pulmonary fibrosis and carrying a telomere-related gene mutation. Eur Respir J. 2021 Feb 1;57(2).

8. Groen K, van der Vis JJ, van Batenburg AA, Kazemier KM, Grutters JC, van Moorsel CHM. Genetic Variant Overlap Analysis Identifies Established and Putative Genes Involved in Pulmonary Fibrosis. Int J Mol Sci. 2023 Feb 1;24(3):2790.

9. van Batenburg AA, Kazemier KM, Peeters T, van Oosterhout MFM, van der Vis JJ, Grutters JC, et al. Cell Type–Specific Quantification of Telomere Length and DNA Double-strand Breaks in Individual Lung Cells by Fluorescence In Situ Hybridization and Fluorescent Immunohistochemistry. J Histochem Cytochem. 2018;66(7).

10. Dhanraj S, Gunja SMR, Deveau AP, Nissbeck M, Boonyawat B, Coombs AJ, et al. Bone marrow failure and developmental delay caused by mutations in poly(A)-specific ribonuclease (PARN). J Med Genet. 2015 Sep 4;52(11):738–48.

11. Kelich J, Aramburu T, van der Vis JJ, Showe L, Kossenkov A, van der Smagt J, et al. Telomere dysfunction implicates POT1 in patients with idiopathic pulmonary fibrosis. J Exp Med. 2022;219(5).

12. Speedy HE, Kinnersley B, Chubb D, Broderick P, Law PJ, Litchfield K, et al. Germ line mutations in shelterin complex genes are associated with familial chronic lymphocytic leukemia. Blood. 2016;128(19):2319–26.

13. Dressen A, Abbas AR, Cabanski C, Reeder J, Ramalingam TR, Neighbors M, et al. Analysis of protein-altering variants in telomerase genes and their association with MUC5B common variant status in patients with idiopathic pulmonary fibrosis: a candidate gene sequencing study. Lancet Respir Med. 2018 Aug 1;6(8):603–14.

14. Van Batenburg AA, Kazemier KM, Van Oosterhout MFM, Van Der Vis JJ, Van Es HW, Grutters JC, et al. From organ to cell: Multi-level telomere length assessment in patients with idiopathic pulmonary fibrosis. PLoS One. 2020;15(1).

15. Ley B, Torgerson DG, Oldham JM, Adegunsoye A, Liu S, Li J, et al. Rare Protein-Altering Telomere-related Gene Variants in Patients with Chronic Hypersensitivity Pneumonitis. Am J Respir Crit Care Med. 2019 Nov 1;200(9):1154–63.

16. Maillet F, Galimard JE, Borie R, Lainey E, Larcher L, Passet M, et al. Haematological features of telomere biology disorders diagnosed in adulthood: A French nationwide study of 127 patients. Br J Haematol. 2024 Nov 1;205(5).

17. Alder JK, Hanumanthu VS, Strong MA, DeZern AE, Stanley SE, Takemoto CM, et al. Diagnostic utility of telomere length testing in a hospital-based setting. Proc Natl Acad Sci U S A. 2018 Mar 6;115(10):E2358–65.

18. Ballew BJ, Yeager M, Jacobs K, Giri N, Boland J, Burdett L, et al. Germline mutations of regulator of telomere elongation helicase 1, RTEL1, in Dyskeratosis congenita. Hum Genet. 2013 Apr;132(4):473–80.

19. Moriya K, Niizuma H, Rikiishi T, Yamaguchi H, Sasahara Y, Kure S. Novel Compound Heterozygous RTEL1 Gene Mutations in a Patient With Hoyeraal-Hreidarsson Syndrome. Pediatr Blood Cancer. 2016 Sep 1;63(9):1683–4.

20. Petrovski S, Todd JL, Durheim MT, Wang Q, Chien JW, Kelly FL, et al. An Exome Sequencing Study to Assess the Role of Rare Genetic Variation in Pulmonary Fibrosis. Am J Respir Crit Care Med. 2017 Jul 1;196(1):82–93.

21. Borie R, Bouvry D, Cottin V, Gauvain C, Cazes A, Debray MP, et al. Regulator of telomere length 1 (RTEL1) mutations are associated with heterogeneous pulmonary and extra-pulmonary phenotypes. Eur Respir J. 2019 Feb 1;53(2):1800508.

22. Zhang D, Newton CA, Wang B, Povysil G, Noth I, Martinez FJ, et al. Utility of whole genome sequencing in assessing risk and clinically relevant outcomes for pulmonary fibrosis. Eur Respir J. 2022 Dec 1;60(6).

23. Niewisch MR, Kim J, Giri N, Lunger JC, McReynolds LJ, Savage SA. Genotype and Associated Cancer Risk in Individuals With Telomere Biology Disorders. JAMA Netw Open. 2024 Dec 11;7(12):e2450111.

24. Speckmann C, Sahoo SS, Rizzi M, Hirabayashi S, Karow A, Serwas NK, et al. Clinical and Molecular Heterogeneity of RTEL1 Deficiency. Front Immunol. 2017 May 1;8(MAY).

25. AM F, L S, C J, NR T, J E, R K, et al. Carrier screening of RTEL1 mutations in the Ashkenazi Jewish population. Clin Genet. 2015 Aug 1;88(2):177–81.

26. Collopy LC, Walne AJ, Cardoso S, de la Fuente J, Mohamed M, Toriello H, et al. Triallelic and epigenetic-like inheritance in human disorders of telomerase. Blood. 2015 Jul 9;126(2):176–84.

27. Keel SB, Scott A, Bonilla MS, Ho PA, Gulsuner S, Pritchard CC, et al. Genetic features of myelodysplastic syndrome and aplastic anemia in pediatric and young adult patients. Haematologica. 2016 Oct 31;101(11):1343–50.

28. Walne AJ, Vulliamy T, Kirwan M, Plagnol V, Dokal I. Constitutional mutations in RTEL1 cause severe dyskeratosis congenita. Am J Hum Genet. 2013 Mar 7;92(3):448–53.

29. Cogan JD, Kropski JA, Zhao M, Mitchell DB, Rives L, Markin C, et al. Rare variants in RTEL1 are associated with familial interstitial pneumonia. Am J Respir Crit Care Med. 2015 Mar 15;191(6):646–55.

30. Jongmans MCJ, Verwiel ETP, Heijdra Y, Vulliamy T, Kamping EJ, Hehir-Kwa JY, et al. Revertant somatic mosaicism by mitotic recombination in dyskeratosis congenita. Am J Hum Genet. 2012 Mar 9;90(3):426–33.

31. van der Vis JJ, van der Smagt JJ, Hennekam FAM, Grutters JC, van Moorsel CHM. Pulmonary Fibrosis and a TERT Founder Mutation With a Latency Period of 300 Years. Chest. 2020 Aug 1;158(2):612–9.

32. Ly H, Schertzer M, Jastaniah W, Davis J, Yong SL, Ouyang Q, et al. Identification and functional characterization of 2 variant alleles of the telomerase RNA template gene (TERC) in a patient with dyskeratosis congenita. Blood. 2005 Aug 15;106(4):1246–52.

33. van der Vis JJ, van der Smagt JJ, van Batenburg AA, Goldschmeding R, van Es HW, Grutters JC, et al. Pulmonary fibrosis in non-mutation carriers of families with short telomere syndrome gene mutations. Respirology. 2021 Sep 27;

34. Borie R, Tabèze L, Thabut G, Nunes H, Cottin V, Marchand-Adam S, et al. Prevalence and characteristics of TERT and TERC mutations in suspected genetic pulmonary fibrosis. Eur Respir J. 2016;48(6):1721–31.

35. Snetselaar R, van Batenburg AA, Van Oosterhout MFM, Kazemier KM, Roothaan SM, Peeters T, et al. Short telomere length in IPF lung associates with fibrotic lesions and predicts survival. PLoS One. 2017;12(12).

36. Planté-Bordeneuve T, Terwiel M, Vis JJ van der, Es W Van, Veltkamp M, Grutters JC, et al. Family history of pulmonary fibrosis impacts prognosis in patients with sarcoidosis. ERJ Open Res. 2024 Oct 4;00441–2024.

37. Newton CA, Batra K, Torrealba J, Kozlitina J, Glazer CS, Aravena C, et al. Telomere-related lung fibrosis is diagnostically heterogeneous but uniformly progressive. Eur Respir J. 2016 Dec;48(6):1710–20.

38. Diaz de Leon A, Cronkhite JT, Katzenstein A-LA, Godwin JD, Raghu G, Glazer CS, et al. Telomere Lengths, Pulmonary Fibrosis and Telomerase (TERT) Mutations. Morty RE, editor. PLoS One. 2010 May 19;5(5):e10680.

39. Schratz KE, Flasch DA, Atik CC, Cosner ZL, Blackford AL, Yang W, et al. T cell immune deficiency rather than chromosome instability predisposes patients with short telomere syndromes to squamous cancers. Cancer Cell. 2023 Apr 10;41(4):807-817.e6.

40. Manali ED, Kannengiesser C, Borie R, Ba I, Bouros D, Markopoulou A, et al. Genotype-Phenotype Relationships in Inheritable Idiopathic Pulmonary Fibrosis: A Greek National Cohort Study. Respiration. 2022 Jun 1;101(6):531–43.

41. Aldera JK, Barkauskas CE, Limjunyawong N, Stanley SE, Kembou F, Tuder RM, et al. Telomere dysfunction causes alveolar stem cell failure. Proc Natl Acad Sci U S A. 2015 Apr 21;112(16):5099–104.

42. Gutierrez-Rodrigues F, Donaires FS, Pinto A, Vicente A, Dillon LW, Clé D V., et al. Pathogenic TERT promoter variants in telomere diseases. Genet Med. 2018;0(0):1–9.

43. Parry EM, Alder JK, Qi X, Chen JJ-L, Armanios M. Syndrome complex of bone marrow failure and pulmonary fibrosis predicts germline defects in telomerase. Blood. 2011 May 26;117(21):5607–11.

44. Sharma A, Myers K, Ye Z, D’Orazio J. Dyskeratosis congenita caused by a novel TERT point mutation in siblings with pancytopenia and exudative retinopathy. Pediatr Blood Cancer. 2014 Dec;61(12):2302–4.

45. Cronkhite JT, Xing C, Raghu G, Chin KM, Torres F, Rosenblatt RL, et al. Telomere Shortening in Familial and Sporadic Pulmonary Fibrosis. Am J Respir Crit Care Med. 2008 Oct 1;178(7):729–37.

46. Gable DL, Gaysinskaya V, Atik CC, Conover Talbot C, Kang B, Stanley SE, et al. ZCCHC8, the nuclear exosome targeting component, is mutated in familial pulmonary fibrosis and is required for telomerase RNA maturation. Genes Dev. 2019 Oct 1;33(19–20):1381–96.

47. Groen K, van der Vis JJ, van Batenburg AA, Kazemier KM, de Bruijn MJW, Stadhouders R, et al. A new variant in the ZCCHC8 gene: diverse clinical phenotypes and expression in the lung. ERJ open Res. 2024 Jan 1;10(1).

48. Schratz KE, Haley L, Danoff SK, Blackford AL, DeZern AE, Gocke CD, et al. Cancer spectrum and outcomes in the Mendelian short telomere syndromes. Blood. 2020 May 28;135(22):1946–56.
